# Statistics

**Published:** 1860-01-01

**Authors:** Frederick Purday

**Affiliations:** Principal of the Statistical Department of the Poor Law Board


The following interesting and valuable statistics are from the remarks of Frederick Purday, Esq., Principal of the Statistical Department of the Poor Law Board, appended to the Parliamentary Return of Insane Paupers chargeable on the first of January, 1859.

1. The statistics of pauper insanity contained in this paper have been compiled from the lists of "Lunatics, Idiots, and other Persons of Unsound Mind," who were chargeable to the poor rates on the 1st January, 1859. These lists are made out annually by the Clerks to the Guardians, and transmitted to the Poor Law Board, in compliance with the 16 and 17 Vict. c. 97, s. 64.

2. In tabulating the numbers under "Lunatic," or "Idiot," this classification has been observed. All cases of congenital insanity, under whatever name returned, have been entered as "Idiots," all the other cases as "Lunatics;" whether the insanity commenced at a stated age or at an "unknown" age.

3. Returns were received from 645* unions and single parishes under Boards of Guardians; the population of these places, according to the census of 1851, was 17,669,448; but, estimated to the 1st January, 1859, it was 19,430,000. The number of insane paupers chargeable to the Poor Rates in those places, on that day, was as follows:—

| Number of Paupers on 1st January, 1859. | Whereof were Insane Paupers. | Namely:— | |
|---|---|---|---|
| | | Lunatics. | Idiots. |
| 867,543 | 30,318 | 21,432 | 8,886 |

Thus 3·50 per cent. of the pauperism, on the 1st January last, is ascribable to insanity; the lunatics being 2·47 per cent., and the idiots 1·03 per cent.

* "Besides these Unions, some places, which do not make the usual returns of Pauperism to the Poor Law Board, have transmitted lists of insane paupers. The total number of insane paupers so returned is 98; viz., 75 lunatics, and 23 idiots; the particulars are printed, with the other Unions, at p. 26, *et seq.*

4. In regard to the sexes, 13,389 were males, and 16,929 females:—

| Insane. | Males. | Females. | Total. |
|---|---|---|---|
| Lunatics | 9280 | 12,152 | 21,432 |
| Idiots | 4109 | 4,777 | 8,886 |
| Total. | 13,389 | 16,929 | 30,318 |

It is worthy of remark that, while the females considerably preponderate among the lunatics, they do not much exceed the males among the idiots. But, taking the two classes together, the ratio of females to males is not so great as it is in the pauperism at large, where, among the adults, the female are more than double the number of the male recipients of relief. The returns of pauperism do not distinguish the sexes of the children, but there is no reason to suppose that, if they were discriminated, the proportion would be materially changed.

5. In the subjoined table, the number of lunatic and idiot paupers is shown for each division of England and Wales.

| Divisions. | Number of Paupers in Receipt of Relief on 1st January, 1859. | Whereof were Insane Paupers. | Namely:— LUNATICS AND IDIOTS. | | | |
|---|---|---|---|---|---|---|
| | | | — | Males. | Females. | Total. |
| I. The Metropolis . . . | 97,707 | 4661 | Lunatics | 1712 | 2507 | 4219 |
| | | | Idiots | 222 | 220 | 442 |
| II. South Eastern . . . | 97,610 | 3207 | Lunatics | 917 | 1271 | 2188 |
| | | | Idiots | 486 | 533 | 1019 |
| III. South Midland . . . | 77,379 | 2298 | Lunatics | 673 | 894 | 1567 |
| | | | Idiots | 354 | 377 | 731 |
| IV. Eastern . . . . . | 76,236 | 2063 | Lunatics | 602 | 830 | 1432 |
| | | | Idiots | 286 | 345 | 631 |
| V. South Western . . . | 107,405 | 3303 | Lunatics | 926 | 1300 | 2226 |
| | | | Idiots | 523 | 554 | 1077 |
| VI. West Midland . . . | 92,706 | 3778 | Lunatics | 1118 | 1403 | 2521 |
| | | | Idiots | 532 | 725 | 1257 |
| VII. North Midland . . . | 49,493 | 2083 | Lunatics | 618 | 721 | 1339 |
| | | | Idiots | 340 | 404 | 744 |
| VIII. North Western . . . | 92,016 | 3370 | Lunatics | 1095 | 1326 | 2421 |
| | | | Idiots | 464 | 485 | 949 |
| IX. York. . . . . . . . | 59,075 | 2233 | Lunatics | 744 | 836 | 1580 |
| | | | Idiots | 293 | 360 | 653 |
| X. Northern . . . . . | 42,712 | 1249 | Lunatics | 396 | 461 | 857 |
| | | | Idiots | 194 | 198 | 392 |
| XI. Welsh . . . . . . | 75,199 | 2073 | Lunatics | 479 | 603 | 1082 |
| | | | Idiots | 415 | 576 | 991 |

6. The ages of 19,886 lunatics are given in the following summary:—

| Years of Age. | Number of Lunatics. | Years of Age. | Number of Lunatics. |
|---|---|---|---|
| Under 5 Years . . . . . . | 4 | 30 Years . . . . . . . | 4400 |
| 5 ,, . . . . . . | 31 | 40 ,, . . . . . . . . | 4638 |
| 10 ,, . . . . . . | 633 | 50 ,, . . . . . . . . | 3659 |
| 20 ,, . . . . . . | 2729 | 60 ,, . . . . . . . . | 2429 |
| | | 70 ,, and upwards . . | 1363 |

7. The ages of 8,792 idiots were as follows:—

| Years of Age. | Number of Idiots. | Years of Age. | Number of Idiots. |
|---|---|---|---|
| Under 5 Years . . . . . . | 10 | 30 Years . . . . . . . | 2012 |
| 5 ,, . . . . . . | 100 | 40 ,, . . . . . . . | 1621 |
| 10 ,, . . . . . . | 1094 | 50 ,, . . . . . . . | 986 |
| 20 ,, . . . . . . | 2159 | 60 ,, . . . . . . . | 537 |
| | | 70 ,, and upwards . . | 273 |

8. In 13,672 cases classed as lunatic, the returns, in stating the length of time "supposed to be of unsound mind," as well as the age of the person, have supplied the means of arriving at the date of the attack. This is shown in quinquennial periods, and in respect of 5923 males, and 7749 females, in the next table.

| Years of Age. | Total. | Males. | Females. | Years of Age. | Total. | Males. | Females. |
|---|---|---|---|---|---|---|---|
| Under 5 Years | 109 | 57 | 52 | 50 Years | 828 | 342 | 486 |
| 5 ,, | 150 | 64 | 86 | 55 ,, | 633 | 239 | 394 |
| 10 ,, | 286 | 132 | 154 | 60 ,, | 478 | 206 | 272 |
| 15 ,, | 912 | 418 | 494 | 65 ,, | 273 | 106 | 167 |
| 20 ,, | 1665 | 777 | 888 | 70 ,, | 171 | 65 | 106 |
| 25 ,, | 1864 | 837 | 1027 | 75 ,, | 91 | 25 | 66 |
| 30 ,, | 1829 | 820 | 1009 | 80 ,, | 34 | 15 | 19 |
| 35 ,, | 1740 | 742 | 998 | 85 ,, | 12 | 4 | 8 |
| 40 ,, | 1407 | 582 | 825 | 90 ,, | 3 | 1 | 2 |
| 45 ,, | 1186 | 491 | 695 | 95 ,, | 1 | | 1 |

9. The 30,318 Insane Paupers were maintained in the following establishments, or were lodged with strangers, or resided with their relatives; namely—

14,481 in County or Borough Lunatic Asylums.
2,076 in Registered Hospitals, or in Licensed Houses.
7,963 in Union or Parish Workhouses.
906 in Lodgings, or Boarded Out.
4,892 Residing with Relatives.

Of the small number returned as "In Lodgings, or Boarded Out," 326, or more than *one-third*, are chargeable to parishes in Wales.

10. The average weekly expense of each insane pauper has been estimated by the respective unions. But it is termed, in conformity to the Schedule, the "Average Weekly Cost of Maintenance and Clothing." This does not appear however to be altogether an accurate description of the expenditure. The cost in county or borough asylums may be illustrated from the example of the Chester Asylum, where, according to the 13th Report of the Commissioners in Lunacy, p. 112, the weekly charge for paupers belonging to the county, or to boroughs in the county, is 8*s*. 2*d*.; for paupers of other counties or boroughs, 14*s*. The cost to the asylum is made up thus:—Provisions, 4*s*. 3*d*.; clothing, 7*d*.; salaries and wages, 2*s*.; necessaries, 9*d*.; surgery and dispensary, 1*d*.; wine, spirits, and porter, 1*d*.; furniture and bedding, 5*d*.; garden and farm, 2*d*.; miscellaneous, 3*d*.; total, 8*s*. 7*d*. The charge in licensed houses will be made up, it may be presumed, of similar expenses, with some addition as the proprietor's profit. The charge in workhouses is the expense of food, clothing, and necessaries, supplied for the in-door paupers.

11. The proportion of Idiots to the total number of Insane Paupers is between *one-third* and *one-fourth*, *i.e*, 29·3 per cent. for the whole country. But there is considerable variation from this proportion in different union-counties, and in different divisions. In the metropolis, where it is the lowest, the ratio is 9·5 per cent.; and in North Wales, where it is highest, it is 52·3 per cent. The following is a list of the divisions, arranged according to the greatest proportionate number of Idiots:—

Ratio per cent. of Idiots on total number of Insane Paupers.

| | | | |
|---|---|---|---|
| Welsh | 47·8 | Northern | 31·4 |
| North Midland | 35·7 | Eastern | 30·7 |
| West Midland | 33·3 | York | 29·2 |
| South Western | 32·6 | North Western | 28·2 |
| South Eastern | 31·8 | The Metropolis | 9·5 |
| South Midland | 31·8 | | |

In *twenty-two* union-counties the ratio *exceeds one-third* of the total of Insane Paupers; namely—

Ratio per cent. of Idiots on total number of Insane Paupers.

| | | | |
|---|---|---|---|
| North Wales | 52·3 | Berks | 36·8 |
| South Wales | 48·8 | Worcester | 36·5 |
| Westmoreland | 44·8 | Bedford | 36·4 |
| Northumberland | 43·6 | Cambridge | 36·3 |
| Derby | 42·2 | Northampton | 35·8 |
| Hereford | 41.5 | Somerset | 35·5 |
| Chester | 40·0 | Stafford | 35·5 |
| Salop | 39.2 | Buckingham | 35·4 |
| Wilts | 37·8 | Huntingdon | 34·5 |
| Nottingham | 37.6 | Hertford | 33·5 |
| Sussex | 37·2 | Lincoln | 33·5 |

12. The ratio of Lunatics, and of Idiots to the total number of Insane Paupers, in the different divisions, is as follows:—

Ratio per cent. of Insane to total Pauperism.

